# Bird-Strike Resistance of Composite Laminates with Different Materials

**DOI:** 10.3390/ma13010129

**Published:** 2019-12-27

**Authors:** Yadong Zhou, Youchao Sun, Tianlin Huang

**Affiliations:** 1 College of Civil Aviation, Nanjing University of Aeronautics and Astronautics, Nanjing 211100, China; 2 Commercial Aircraft Engine Co., Ltd., Aero Engine Cooperation of China, Shanghai 201100, China

**Keywords:** bird strike, composite materials, damage mode, impact energy, modal frequency

## Abstract

To obtain some basic laws for bird-strike resistance of composite materials in aeronautical application, the high-velocity impact behaviors of composite laminates with different materials were studied by numerical methods. The smoothed particle hydrodynamics (SPH) and finite element method (FEM) coupling models were validated from various perspectives, and the numerical results were comparatively investigated. Results show that the different composite materials have relatively little effect on projectile deformations during the bird impact. However, the impact-damage distributions can be significantly different for different composite materials. The strength parameters and fracture energy parameters play different roles in different damage modes. Lastly, modal frequency was tentatively used to explain the damage behavior of the composite laminates, for it can manifest the mass and stiffness characteristics of a dynamic structure. The dynamic properties and strength properties jointly determine the impact-damage resistance of composite laminates under bird strike. Future optimization study can be considered from these two aspects.

## 1. Introduction

In civil aviation industry, bird-strike accidents are a serious threat to airplane passengers and crew. A number of bird-strike accidents occur every year, and many of them are due to the collision with wing leading edges or windshields or the ingestion of birds into aero-engines, which has a higher probability than with other front-facing components. Bird-strike impacts are the major cause of blade damage for aircraft engines [1]. Therefore, the bird-induced failure of both fan blades [2,3,4,5] and leading edges [6,7,8,9,10,11] have caused much research interest. The latest bird-strike example was an Airbus A321 of Russian Ural Airlines on 15 August 2019, which was struck by birds during take-off, and subsequently its dual engines failed. The airworthiness regulations of the Federal Aviation Administration (FAA), European Aviation Safety Agency (EASA), and Civil Aviation Administration of China (CAAC) have all defined the requirements of anti-bird impact for aircraft structures [12].

To improve the aerodynamic performance and structural efficiency, material systems for aero-engine fan blades and leading edges are transferring from alloys to composites. Consequently, it is highly important to assess and optimize the bird-strike resistance of composite materials for aeronautical applications. The knowledge of high-velocity impact resistance of composite material systems is highly required and has aroused much attention in recent years. Grimaldi et al. [13] studied the effect of different through-thickness lay-up configurations on bird-impact energies of laminates. Hedayati et al. [14] investigated five types of lay-ups to calculate each critical thickness preventing the bird from perforating. Hedayati et al. [15] showed that the optimum inner plate position was inserted at the middle of the foam in a sandwich structure under bird strike. Mohagheghian et al. [16] showed that the order of the glass layers can significantly influence the high-velocity soft impact performance. Mohagheghian et al. [17] considered the choice of the polymer interlayer for laminated aircraft windshields. Kaboglu et al. [18] studied the high-velocity impact properties of various types (different stacking configurations and thicknesses) of fiber metal laminates, showing perforation resistance was significantly improved with increasing the number of alternating layers. Guida et al. [6] hybridized carbon fiber reinforced plastic laminates with shape memory alloy materials and showed better impact properties of the laminates containing the hybrid layers. Di Caprio et al. [7] considered the bird-strike crashworthiness of different material systems (different stacking sequences and total thicknesses), without changing the overall geometrical shape. Liu et al. [19] experimentally studied the damage of hygrothermally conditioned carbon epoxy composites under high-velocity impact. Wagner et al. [20] experimentally and numerically assessed different aerospace-grade composite materials under high-velocity impact. Sun et al. [21] simulated the eccentric impact of square and rectangular composite laminates embedded with shape-memory-alloy wires. Liu et al. [22] experimentally observed matrix cracking and fiber breakage at the high velocity of 100 m/s on the rear surface of the impacted carbon/epoxy composite. These abovementioned works have indicated that the selection of lamina materials is a key issue and have shown promising results in material modification of composite laminates for bird-strike resistance. Authors have recently investigated some critical influencing factors for bird-strike damage of laminated composite materials, such as the total laminate thickness [23], ply angles [24], rotational speeds [25], and impact locations/configurations [26,27]. However, the influence of different composite materials has not been considered very well.

As Ali et al. [28] pointed out, developing the improved and tough composite materials to lessen severity of impact damage has been a major challenge for researchers. It is well-known that impact resistance of different materials will be different, and it should be considered that the total thickness is frequently a limit for critical aircraft structures. However, some generic laws for anti-bird-strike ability are still not studied very well. Therefore, in the present article, different lamina materials with a fixed total thickness are considered to compare the impact properties so as to obtain some generic laws for bird-strike resistance of composite materials.

## 2. Numerical Model

At high velocities, the bird projectile behaves like fluidic materials, and its inhomogeneity becomes negligible and hence can be treated as “soft body” [29,30]. The hydrodynamic responses of fluidic materials can be accurately modelled by the smoothed particle hydrodynamics (SPH) method, which has experienced wide use in bird-strike simulations [2,25,29,31,32,33,34]. In the present simulation study with ABAQUS software, the bird projectile was firstly meshed by finite elements (C3D8R), and then they were converted to SPH particles (PC3D) at the beginning of the bird-impact analysis (time threshold = 0). A cylinder-shaped projectile was considered, with the length-to-diameter ratio being 2 (length 200 mm and diameter 100 mm, respectively). The mass density of the bird projectile was 950 kg/m^3^, and the total mass was 1.49 kg. The Mie–Grüneisen equation of state (EOS) was used to model the hydrodynamic behavior of the bird projectile. This type of EOS is linear in energy and assumes a linear relationship between the shock velocity (*U_s_*) and the particle velocity (*U_p_*), which provides the linear *U_s_* − *U_p_* Hugoniot form. In the linear Hugoniot form, the relationship *U_s_* = *c*_0_ + *sU_p_* is determined by the parameters *c*_0_ (the sound velocity in the projectile) and *s* (material property). For gelatin projectiles [35], *c*_0_ = 1483 m/s, and material properties *s* = 0 and *Γ*_0_ = 0 were used in ABAQUS modelling.

Dealing with the impact damage of composite laminates, the continuum damage mechanics (CDM) can well describe the intra-laminar damage [36] and have been widely adopted [7,33,37,38] in the framework of finite element method (FEM). The square laminate was modelled by CDM in present study. The in-plane size was 500 mm × 500 mm, and the total thickness was 3.6 mm (0.15 mm × 24 mm). In the global coordinate, the area centroid of the plate corresponds to the center of the projectile’s front surface. The boundary condition was the encastre case on the four edges; that is, the perimeter of the plate was clamped, and thus all degrees of freedom (DOFs) of the four edges were constrained. Three different composite material systems, generally used for aeronautical applications, were investigated: (1) T700/M21 carbon/epoxy material, (2) M91/IM7 carbon/epoxy material, (3) lamina properties from reference [33], which was the carbon fiber reinforced plastics composites. The material properties of the first two materials (T700/M21 and M91/IM7) were taken and simplified from the reference [36]. Table 1 gives the material properties of the three laminas, containing four sections: Mass density (*ρ*), elasticity (*E*, *G*, *ν*), strength (*X*, *Y*, *S*), and fracture energy (*G*c). Mechanical behavior of composite materials under high-velocity impact shows brittle features, and the plasticity can be neglected. The orthotropic elasticity and Hashin’s damage criteria were applied in the explicit dynamic analysis, which can model the initiation of four different types of impact damage: Fiber/matrix tension/compression modes.

Contact was defined between the impacted laminate and the bird projectile. The contact includes surface pairs of all exterior faces. In the impact contact process, the particles behave as spheres. The frictionless tangential behavior and hard contact of normal behavior were defined for the contact properties.

**Figure 1:**
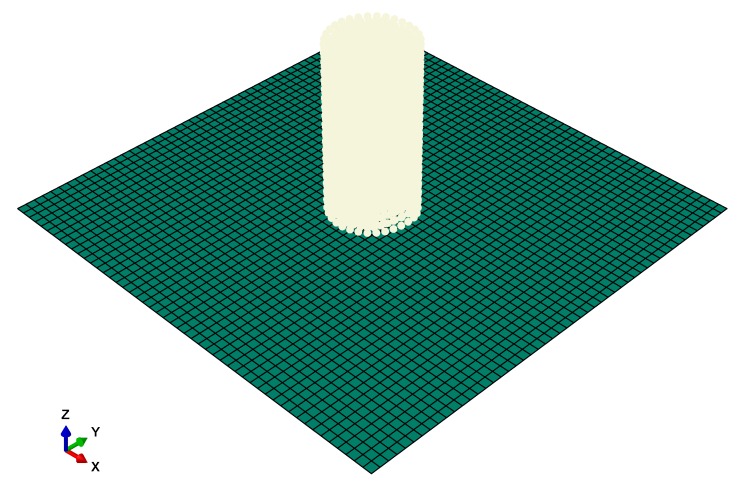
Numerical model of the bird projectile and the square laminate. Finite element method (FEM) meshes are indicated by the dark cyan, smoothed particle hydrodynamics (SPH) particles are indicated by the greyish white.

The laminate’s layups of the three plates are [0/45/0/−45/−45/0/45/0]_3_. The stacking sequence in the square brackets gives the basic eight layups, and the total through-thickness layups are three copies of it, i.e., 24 plies in total. Figure 1 shows the numerical model of the bird projectile and the square laminate. For the impacted plate, 2500 linear quadrilateral elements of type S4R were used, and the total number of nodes was 2601. For a structural level simulation, typical element sizes of 5 mm were performed [20]. A preliminary study was conducted, which validated that the present meshing density (10 mm) was reasonable for comparison purpose, so that the computation efficiency could also be ensured. For the bird projectile, 2220 linear hexahedral elements of type C3D8R were used, and the total number of nodes was 2688. These nodes were converted into SPH particles (PC3D) during the explicit dynamic analysis. The initial velocity was 200 m/s along the *z* direction, i.e., vertical to the plate.

## 3. Results and Discussion

**Figure 2:**
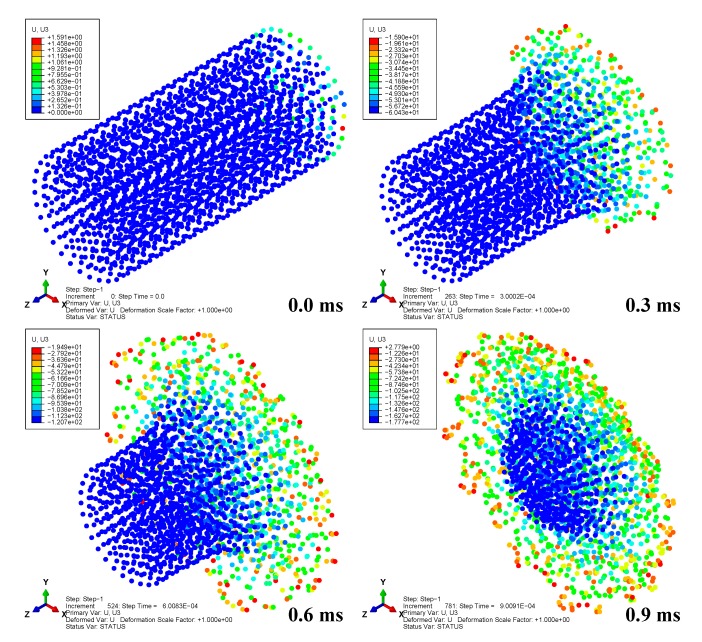
Deformation series of the bird projectile impacting the plate with material 1.

**Figure 3:**
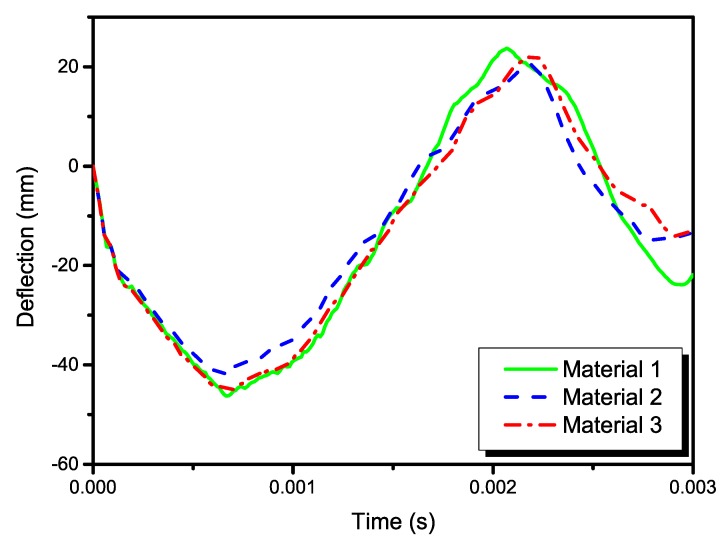
Deflection histories at the plate centers with the three different materials.

**Figure 4:**
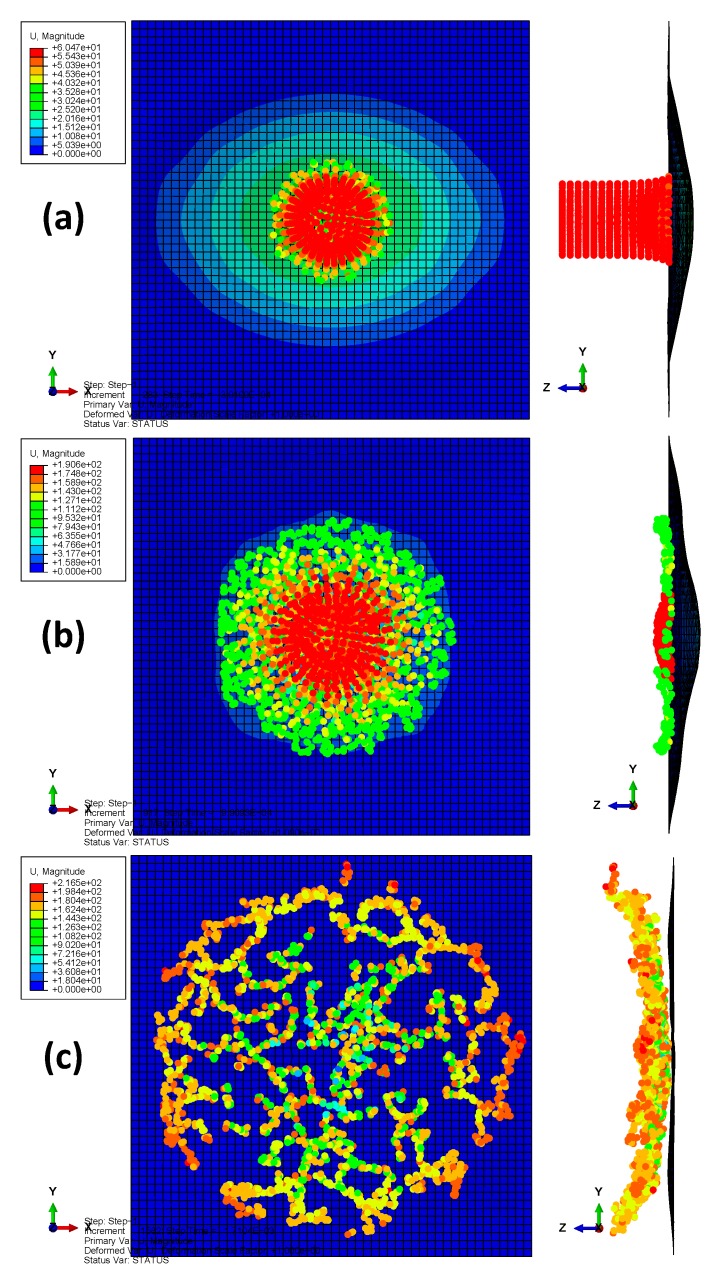
Projectile deformation series in the model of laminate with material 3: (**a**) 0.3 ms; (**b**) 1.0 ms; (**c**) 1.7 ms.

**Figure 5:**
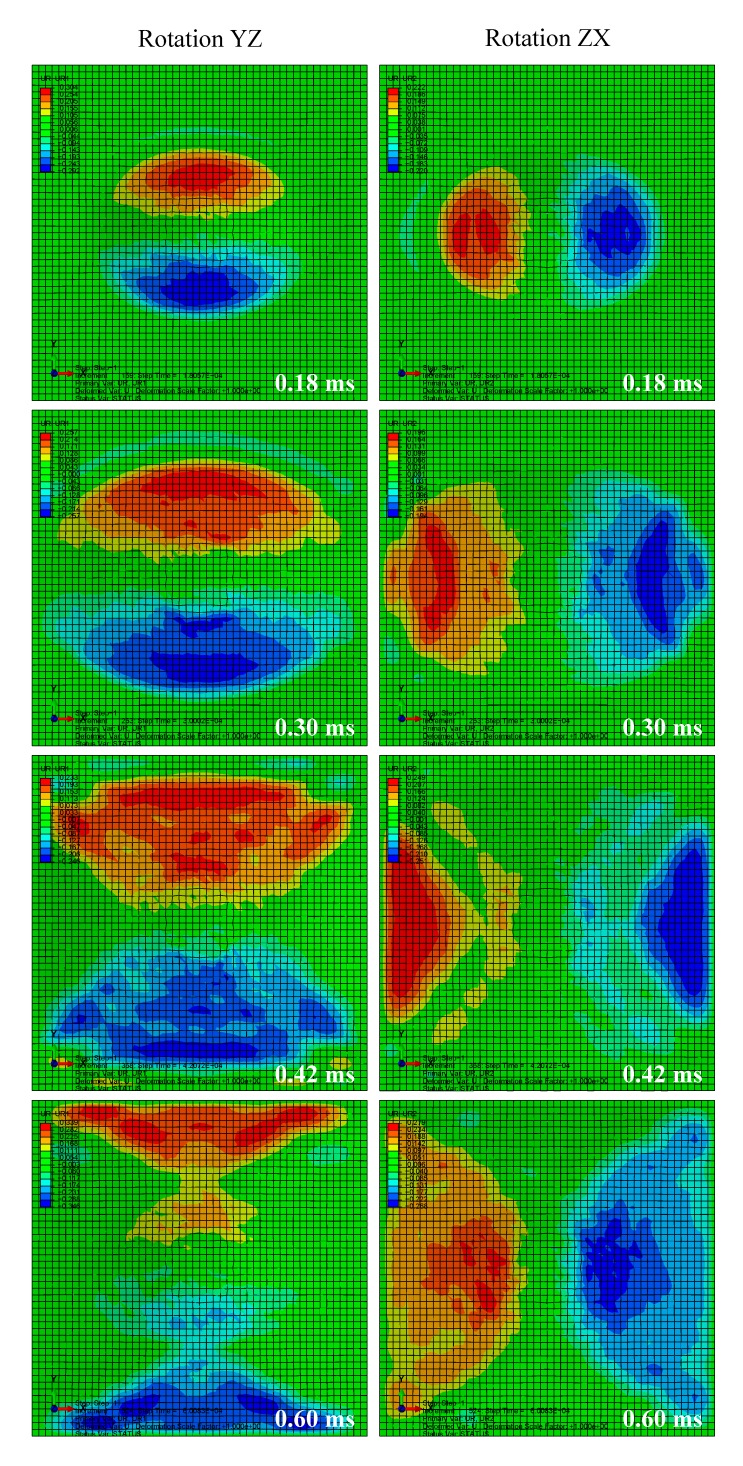
Rotational deformations of the laminates with material 1.

A total time period of 10 ms was simulated. Figure 2 depicts the deformation series of the bird projectile impacting the plate with material 1, with the contour denoting the displacement along *z*-direction. It can be seen that the deformation series agree well with the experimental results in previous literature for high-velocity soft-body impacts. The SPH particles firstly contacted the target and then spread radially over the surface. Results in Figure 2 can also indicate the rationality of the particle density of the bird projectile. Figure 3 depicts the deflection histories at the plate centers with the three different materials. From the time histories, it can be observed that the three plates experienced the maximum deflections at approximately 0.6 ms, and then the plates began to rebound. Figure 4 depicts the global magnitude of the displacements for both the bird particles and the plate with the material system 3 at three different time steps: (a) 0.3 ms; (b) 1.0 ms; (c) 1.7 ms, and both the front and side views are presented to further observe the impact deformations. The impact contact began at 0 ms. It can be seen that the projectile firstly contacted the plate, and then started to spread onto the plate surface, of which the deformation processes are similar to the previous experimental observation of soft-body impact on flat plate with other materials [7,39]. At 1.7 ms approximately, the plate began to return the neutral plane (the original undeformed plane), and the dispersed debris of the projectile began to depart the plate surface. At this point in time, the deformation of the plate was relatively small. Because of the lack of any 90° ply in the stacking sequence, the laminate was not quasi isotropic but still maintained the symmetry. In order to highlight the effect of not having any ply at 90°, the rotational deformations in *yz* and *xz* directions are studied, as shown in Figure 5. The rotational deformations extended from the center to the boundaries during the impact. It can be observed that the rotational deformations exhibit a symmetrical feature in each direction, which can be ascribed to the axial symmetry of the stacking sequence.

**Figure 6:**
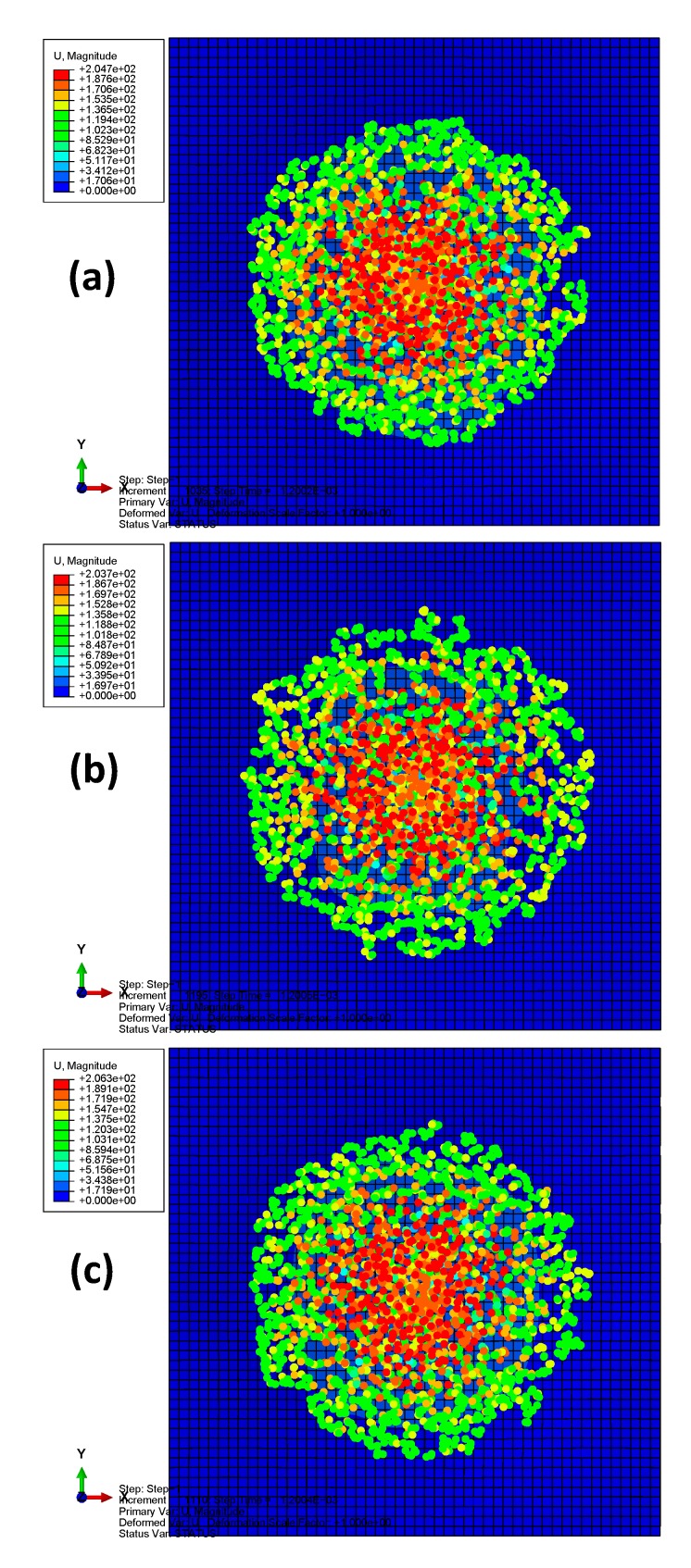
Projectile deformations at 1.2 ms in the models with different plate’s materials: (**a**) material 1; (**b**) material 2; (**c**) material 3.

**Figure 7:**
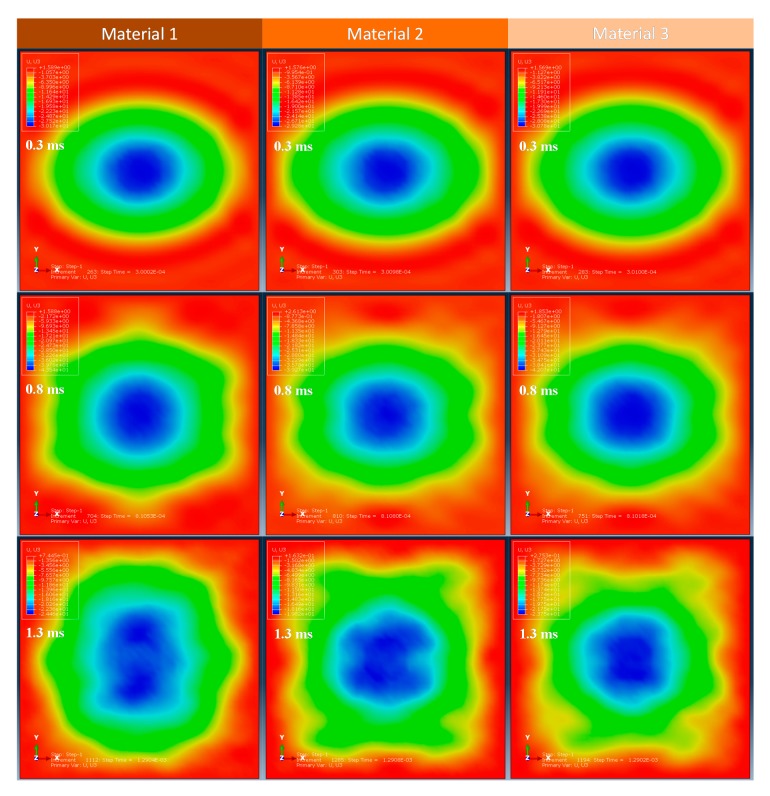
The out-of-plane (U3) displacements of the plates with different material systems.

According to the numerical results, the projectile deformations of the three models are similar to each other, as shown in Figure 6, which depicts the projectile deformations (magnitudes of the particle displacement) at 1.2 ms in the models with different plate’s materials. Therefore, the different composite materials have little effect on projectile deformations. This fact can be further indicated in the time histories of the deflections, as shown in Figure 3. Before the plates go back to the neutral position, the time histories of the deflections at the plate’s centers are quite similar to each other. During this stage, the projectile contacted on the plate surface. Thus, the projectile deformations are also similar. Furthermore, in order to highlight the deformation differences among the analyzed plate models, Figure 7 compares the out-of-plane displacements for the plates at different time steps, which can provide global information of the structural impact response. Results can also verify that the global displacements were quite similar in the square laminated plates made of different material systems.

**Figure 8:**
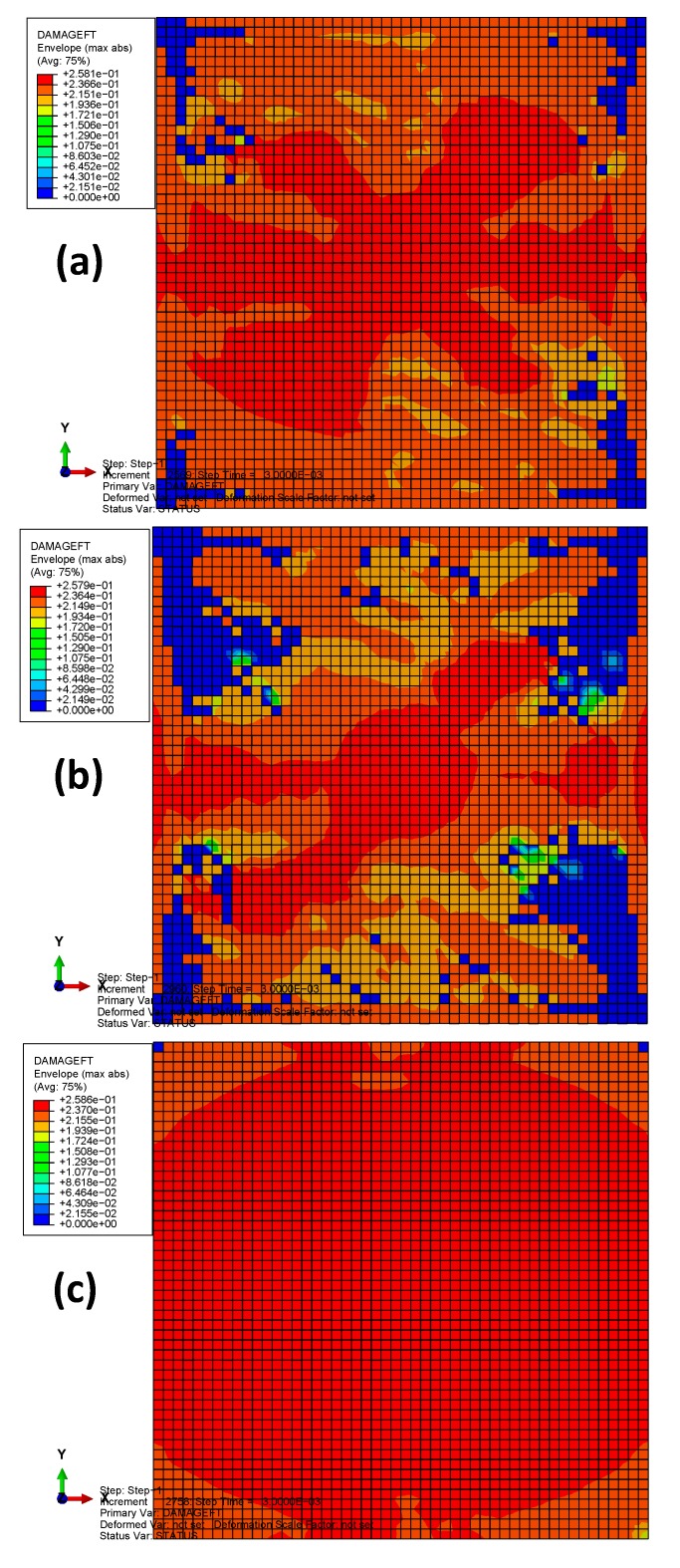
The fiber tension damages of the three models after bird impact: (**a**) material 1; (**b**) material 2; (**c**) material 3.

**Figure 9:**
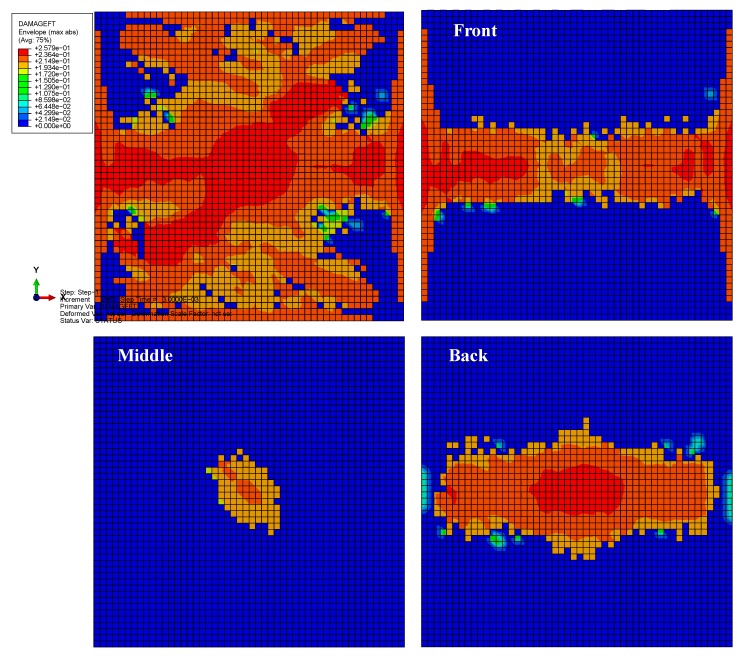
The fiber tension damages at different plies of the plate with material 2.

The envelope quantity was used to compare the impact damages of the different material systems, i.e., the envelope damage of the 24 plies in total. Figure 8 depicts the fiber tension damages (the term DAMAGEFT reported in the legend) of the three models after bird impact. The colors from blue to red indicate the damage states ranging from the undamaged to the maximum damaged. If the damage index of an element equals 1, this element has completely lost its load-carrying capacity. The damaged area of the laminate with material 3 is the largest (≈100%), while that of material 2 is the smallest (≈80%). The reason can be ascribed to the different properties of the fiber tension strength *X*_T_, for which material 2 is the best and material 3 is the worst. Therefore, the fiber tension damage is mainly governed by the fiber tension strength, while the longitudinal tensile fracture energy $G_{1c}^{T}$ has little effect on it because $G_{1c}^{T}$ of material 3 is approximately 23 times higher than that of materials 1 and 2. Figure 9 depicts the fiber tension damages at different plies (front, middle, and back plies) of the plate with material 2. Results show that both the center of the back side and the boundary of the front side experience relatively high fiber-tension damage, while the middle ply experiences relatively small damage. These distribution laws agree well with the experimental observations from the previous literature dealing with the other composite material systems under high-velocity impact [16,22,40].

**Figure 10:**
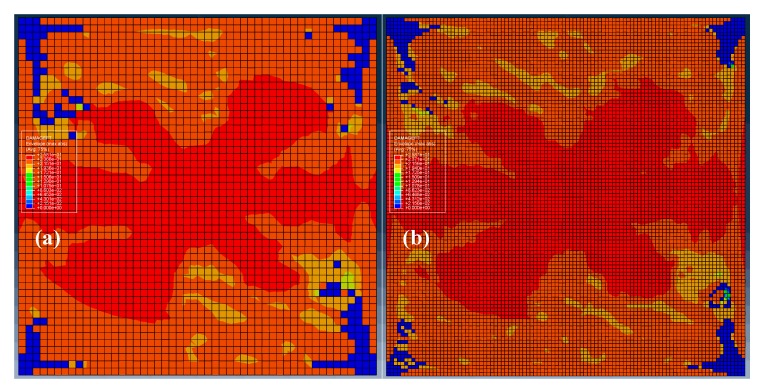
The comparison of fiber-tension damages in the laminate (material 1) with different meshes: (**a**) relatively coarse mesh, (**b**) dense mesh.

In terms of failure analysis of composite materials, it requires the mesh size to be small enough in order to well capture the fracture properties, but in the explicit dynamic analysis of structures under impact, the mesh size cannot be too small, so as to avoid the numerical instability. The reason is that the stability limit Δ*t_stable_* is approximately proportional to the smallest element dimension in the mesh, which can be expressed as $\Delta t_{stable}\approx L_{\min}/C_{d}$, where *L*_min_ is the smallest element dimension in the mesh, and *C_d_* is the wave speed of the material. In brief, in the non-linear simulation of structural impact, we should strike a balance between computational burden and numerical accuracy. To demonstrate present meshes applicable for the damage analysis, Figure 10 compares the fiber-tension damages of the laminate with material 1 by using different meshes. It can be validated that the relatively coarse meshes are applicable to predict the damage distributions.

**Figure 11:**
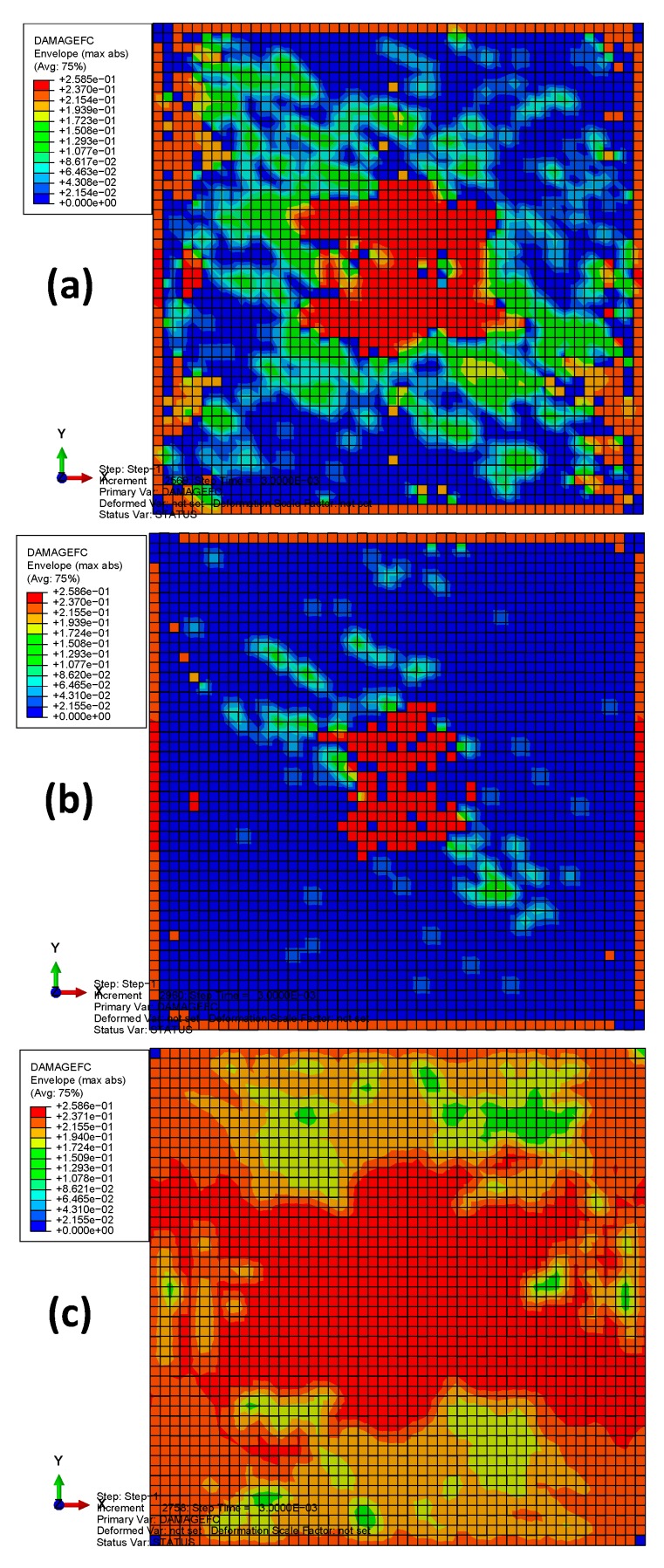
The fiber compression damages of the three models after bird impact: (**a**) material 1; (**b**) material 2; (**c**) material 3.

Figure 11 depicts the fiber compression damages of the three models after bird impact. It can be seen that the damage distributions in materials 1 and 2 are similar to each other (located at the center and the boundaries of the plate), but material 1 has a larger damaged area, mainly due to the lower value of fiber compression strength *X*_C_. Results also show significant difference between the fiber compression damage distributions of materials 1, 2, and that of material 3. The reason can be ascribed to the relatively lower longitudinal compressive fracture energy ($G_{1c}^{C}$) of material 3. Therefore, the compressive strength parameters can be considered to determine the severity of fiber compression damage, while the compressive fracture energy determines the damage distribution patterns.

**Figure 12:**
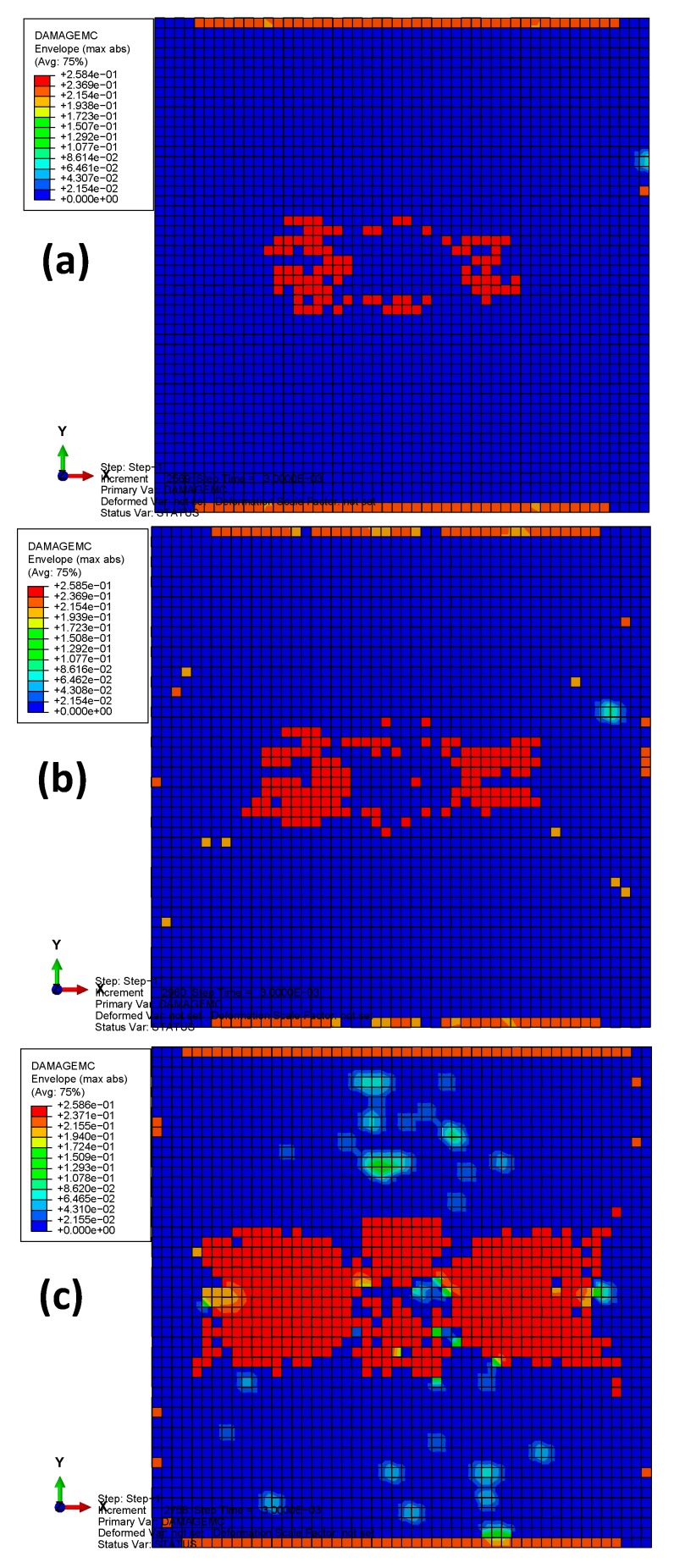
The matrix compression damages of the three models after bird impact: (**a**) material 1; (**b**) material 2; (**c**) material 3.

Figure 12 depicts the matrix compression damages of the three models after bird impact. It can be seen that the damage distributions in the three materials are similar to each other (located in the area around the initial impact contact). However, results show significant increase in the matrix compression damage of material 3. From the initiation criteria of Hashin’s damage, it can be seen that the matrix compression damage mainly depends on the longitudinal shear strength (*S*_12_) and the transverse shear strength (*S*_13_) when transverse compression strength (*Y*_C_) is higher than the transverse shear strength (*S*_13_). Herein, both *S*_12_ and *S*_13_ of materials 1 and 2 are higher than those of material 3. Consequently, the most severe matrix compression damage appeared in material 3.

**Figure 13:**
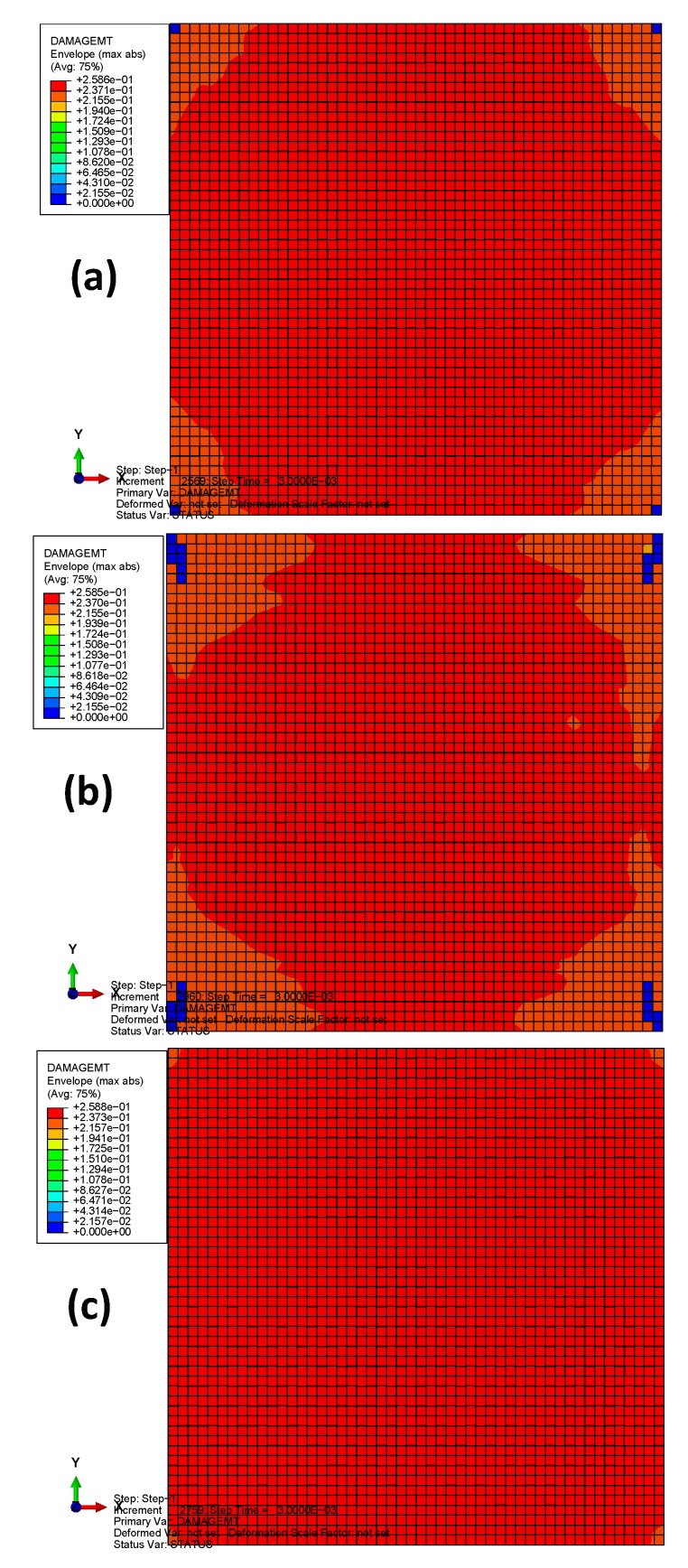
The matrix tension damages of the three models after bird impact: (**a**) material 1; (**b**) material 2; (**c**) material 3.

Figure 13 depicts the matrix tension damages of the three models after bird impact. It can be observed that the damage distributions are similar on the whole, and relatively wide damaged area appeared in the model with material 3. In the damage initiation criteria, the transverse tensile strength (*Y*_T_) and the longitudinal shear strength (*S*_12_) are the beneficial factors for the matrix compression damage. Comparing the material properties, it can be concluded that *Y*_T_ and *S*_12_ are the most influential factors for matrix tension damage, while the tensile fracture energies have little influence.

**Figure 14:**
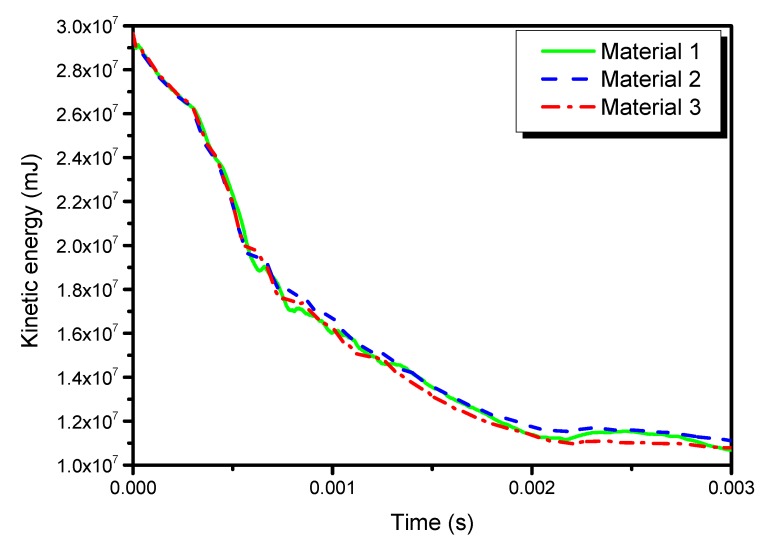
Time histories of the kinetic energy in the three models.

**Figure 15:**
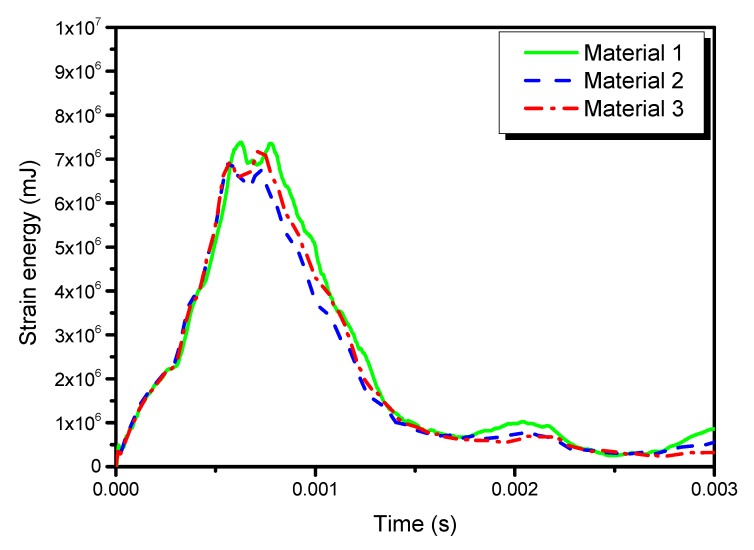
Time histories of the strain energy in the three models.

**Figure 16:**
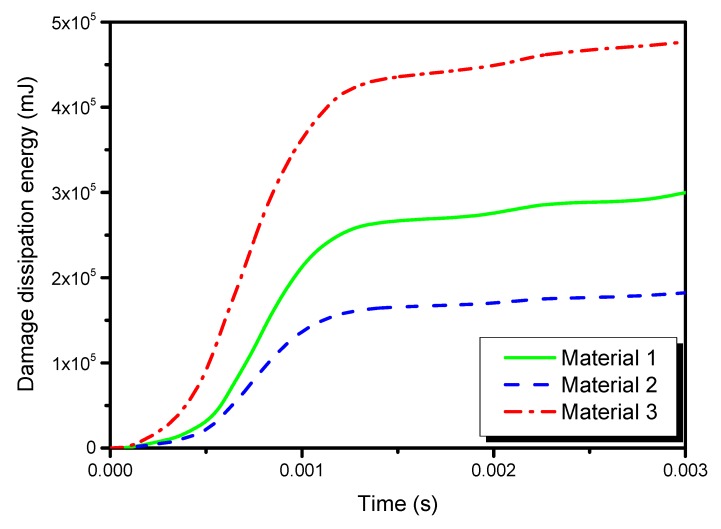
Time histories of the damage dissipation energy in the three models.

Figure 14, Figure 15 and Figure 16 show the time histories of kinetic energies, strain energies, and damage dissipation energies in the three models, respectively. The time histories of kinetic energies and strain energies in the three models are close to each other, which also indicates that the global deformations are similar in different materials. However, the damage dissipation energies are quite different in the three models, with material 2 the lowest and material 3 the highest. A preliminary explanation is that material 2 is characterized by high stiffness and high strength. On the contrary, it can be noted that material 3 is characterized by the lowest strength parameters.

**Figure 17:**
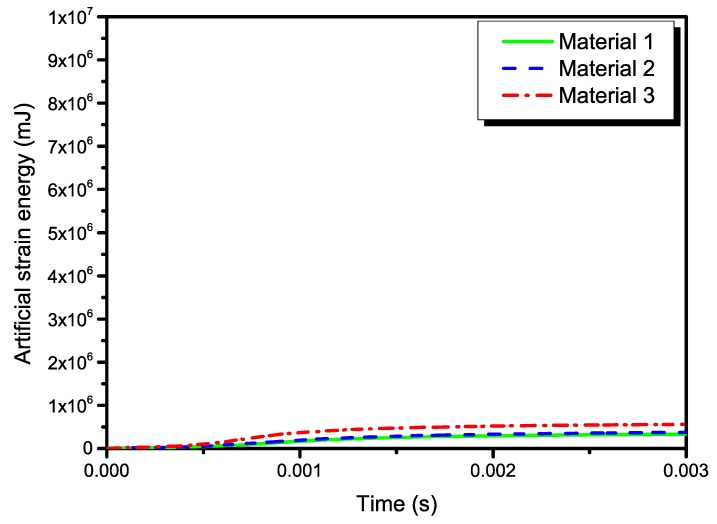
Time histories of the artificial strain energy in the three models.

**Figure 18:**
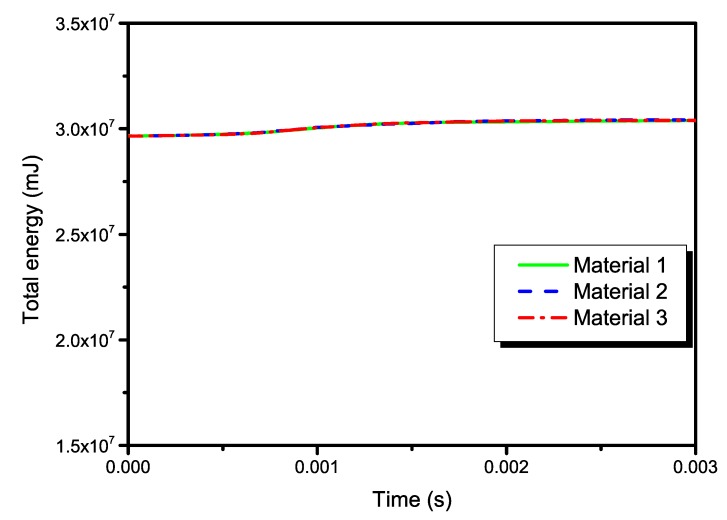
Time histories of the total energy in the three models.

Energy output is particularly important in checking the accuracy of the solution in an explicit dynamic analysis. In order to further validate the global quality of the numerical simulations, the artificial strain energies of the three models are plotted in Figure 17, which belongs to a kind of “artificial” energy and should be negligible compared to “real” energies such as the strain energy and the kinetic energy in explicit dynamic analysis. Theoretically speaking, the total energy in explicit dynamic analysis should be a constant, i.e., the total energy balance. In numerical computation, it can usually be approximate to a constant, as long as the error is small enough. The results in Figure 18 indicate that the total energies of present simulations are close to a constant. From Figure 14, Figure 15, Figure 16, Figure 17 and Figure 18, the effectiveness of present numerical simulations can also be verified.

**Figure 19:**
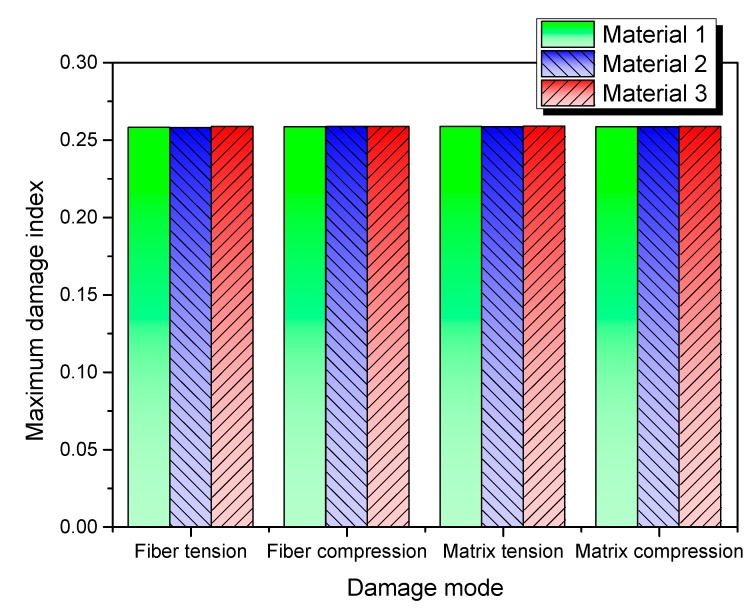
Maximum local damage indices for different damage modes in the three models.

Figure 19 shows the maximum damage indices for different damage modes in the three models. It can be seen that local maximum damages are almost identical for the four damage modes of the considered three materials. This phenomenon can be ascribed to the same structural configurations and the identical input impact energy (*E*_impact_) considered in present simulations. The bird-strike impact energy (*E*_bird-strike_) is defined by the impact velocity and projectile mass as *E*_bird-strike_ = 0.5 × *m*_bird_ × $V_{\mathrm{relative}}^{2}$, where *m*_bird_ is the mass of bird and *V*_relative_ is the relative velocity between the impacted structure and the projectile. In present simulations, *E*_bird-strike_ approximately equals 29.65 kJ. In the considered impact duration, the total energy absorption (*E*_absorption_) of the impacted plate can be determined from the equation $E_{\mathrm{absorption}}=\sum_{N}\left(\frac{1}{2}mV_{\mathrm{relative}}^{2}-\frac{1}{2}mV_{\mathrm{residual}}^{2}\right)$, where *m* is the mass of SPH particle; *V*_residual_ is the residual velocity; and the symbol ∑ depicts the sum of all SPH particles (*N*). The initial impact energy of the projectile was partly absorbed by the plate and partly carried by the particles (residual velocity). The former is mainly in the form of deformation and damage; the latter is mainly in the form of the residual velocities of each projectile particle and the vibrating plate.

From the damage distributions and the local maximum damage, some similar laws can be found with the experimental results of previous literature. Wagner et al. [20] have concluded that different carbon fiber composites have very similar ballistic limits but differ in terms of delamination behavior. In present simulations, the local maximum damage induces are highly similar, but the damage areas differ greatly for different composite materials.

**Figure 20:**
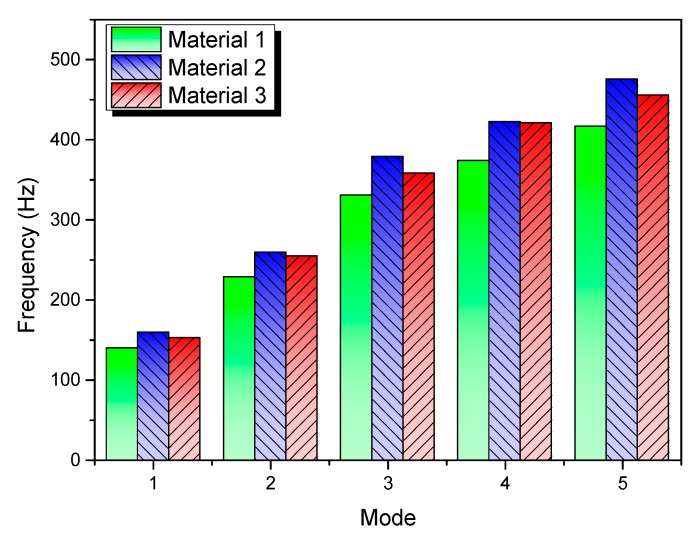
Modal frequencies of the plates with different materials.

Lastly, considering the fact that modal parameters are greatly useful to characterize the failure resistance under dynamic loadings [41,42], modal analysis was conducted to possibly explain the damage results. Structural modal behaviors are determined by the mass matrix (M) and stiffness matrix (K) together in FEM format. As indicated in the multiple degree-of-freedom (*n*) system, the frequency equation $\det\left(K-\omega^{2}M\right)=0$ can determine the characteristic values (*ω*_1_, *ω*_2_, ∙∙∙, *ω*_n_) and can hence reveal the inherent dynamic properties of a system. The matrix $K-\omega^{2}M$ is called dynamic stiffness when structural damping can be neglected, which is the case for high-velocity impact. Modal frequencies can be considered as an indicator to evaluate the mass distribution and the equivalent stiffness. Table 2 gives the total masses and fundamental frequencies of the three plates. Figure 20 compares the first five modal frequencies of the three plates with different materials. Therefore, from the viewpoint of dynamics, it can be seen that material 2 has the better stiffness properties than the other two. In addition, in spite of the largest mass and lowest frequencies, the laminate of material 1 has better impact resistance than that of material 3, mainly due to the higher strength properties. In brief, the dynamic properties and strength properties jointly determine the impact damage resistance of composite laminates under bird strike. Future optimization study can be considered from these two aspects.

## 4. Conclusions

By means of SPH-FEM modelling, this study numerically investigated the bird-impact behaviors (deformations and in-plane damages) of composite laminates with three different materials for aeronautical applications. The numerical models were validated from various perspectives, and the numerical results were comparatively investigated in detail. The following conclusions can be drawn:

1. Results show that the different composite materials (with the same reinforced fibers) have little effect on projectile deformations during the bird impact;
2. The fiber tension damage is mainly governed by the fiber tension strength, while the longitudinal tensile fracture energy has little effect on it;
3. The compressive strength parameters determine the severity of fiber compression damage, while the compressive fracture energy determines the damage distribution patterns;
4. The three composite materials have similar distribution patterns in the matrix compression damage, while the most severe matrix compression damage appeared in material 3, due to its weakest shear strengths;
5. The parameters *Y*_T_ and *S*_12_ are the most influential factors for the matrix tension damage, while the tensile fracture energies have little influence;
6. Modal frequency was tentatively used to explain the damage behavior of the composite laminates, for it can manifest both the mass and stiffness characteristics of a dynamic structure. The dynamic properties and strength properties jointly determine the impact damage resistance of composite laminates under bird strike.

## Figures and Tables

**Table 1:** Material properties of the considered laminas.

Parameter	Unit	T700/M21	M91/IM7	Reference [33]
*ρ*	kg/m^3^	1600	1570	1400
*E*_11_	GPa	130	170	130.05
*E*_22_	GPa	7.7	8.8	11.55
*G*_12_	GPa	4.8	5.5	6
*G*_13_	GPa	4.8	5.5	6
*G*_23_	GPa	4.8	5.5	6
*ν*_12_		0.33	0.228	0.312
*X*_T_	MPa	2080	2700	1022.7
*X*_C_	MPa	1100	1590	613.5
*Y*_T_	MPa	60	105	54
*Y*_C_	MPa	180	252	170
*S*_12_	MPa	110	105	63
*S*_13_	MPa	110	105	63
$G_{1c}^{T}$	kJ/m^2^	0.5	0.5	11.48
$G_{2c}^{T}$	kJ/m^2^	2.1	2.1	4.13
$G_{1c}^{C}$	kJ/m^2^	0.5	0.5	0.35
$G_{2c}^{C}$	kJ/m^2^	2.1	2.1	3.23

**Table 2:** The total masses and modal frequencies of the three plates.

Material System	Material 1	Material 2	Material 3
**Total Mass (kg)**	1.44	1.41	1.26
**Fundamental Frequency (Hz)**	139.28	159.09	152.25

## Data Availability

The data used to support the findings of this study are available from the corresponding author upon request.
